# A successful case of electrical storm rescue after acute myocardial infarction

**DOI:** 10.1186/s12872-022-02982-2

**Published:** 2022-12-09

**Authors:** Bin Liu, Bo Xie, Xun Chen, Ke Zhu, Cheng-Ming Wang, Shu-Hong Guo

**Affiliations:** 1 First Division, Department of Respiratory and Critical Care Medicine, Zhuzhou Hospital, Affiliated to Xiangya School of Medicine, Central South University/Zhuzhou Central Hospital, Zhuzhou, 412007 Hunan China; 2 Medical Affairs Department, Zhuzhou Hospital, Affiliated to Xiangya School of Medicine, Central South University/Zhuzhou Central Hospital, Zhuzhou, 412007 Hunan China; 3 Cardiovascular Medicine Department, Zhuzhou Hospital, Affiliated to Xiangya School of Medicine, Central South University/Zhuzhou Central Hospital, Zhuzhou, 412007 Hunan China

**Keywords:** Electrical storm (ES), Myocardial infarction (MI), Esmolol, Percutaneous coronary intervention (PCI), Thrombus

## Abstract

**Background:**

Electrical storm (ES) is a heterogeneous clinical emergency that can present with malignant ventricular arrhythmias such as ventricular fibrillation (VF), ventricular tachycardia (VT), requiring the need for cardiac defibrillation. ES is a life-threatening condition with a high mortality rate. Successfully managing ES in the setting of acute myocardial infarction (MI) is expected to be known by physicians on call to reduce in-hospital mortality.

**Case presentation:**

A 57-year-old man presenting with acute onset chest pain was found to have an infero-posterior ST-segment elevation myocardial infarction (STEMI) complicated by acute right ventricular MI secondary to total occlusion of the proximal right coronary artery (RCA). The patient developed ES in the form of recurrent VF that was managed successfully with electrical defibrillation, antiarrhythmic therapy with amiodarone and esmolol, endotracheal intubation, sedation, electrolyte replacement, volume resuscitation, comfort care, psychological intervention, and percutaneous coronary intervention (PCI) of the occluded epicardial artery. With these interventions used in quick succession and with the aspiration of a massive RCA thrombus, the patient was reversed to hemodynamic stability, did not have further episodes of VF, and survived the index hospitalization.

**Conclusion:**

ES is a rare but fatal complication of acute MI. Residents on night shifts should be better prepared and equipped to deal with this rare condition. We hope our successful experience can benefit physicians on call who take care of acute MI patients that deteriorate with ES.

## Background

Electrical storm(ES) is defined as three or more protracted episodes of ventricular fibrillation(VF) or presence of hemodynamically unstable ventricular tachycardia (VT) occurring within 24 h, usually requiring electrical defibrillation or electrical cardioversion [1], and this definition has been widely adopted. Electrical defibrillation and cardioversion are the two primary interventions in restoring hemodynamic stability, but overzealous intervention can cause myocardial damage, leading to progressive heart failure and arrhythmias [2]. Therefore, the management of ES should be aimed at addressing the inciting factors and removing the potential triggers and improving the prognosis through timely percutaneous coronary intervention (PCI) to open vascular occlusions and the use of effective antiarrhythmic drugs, as well as considering the appropriate use of analgosedation.

ES carries a very high fatality rate, especially if it occurs after acute coronary syndrome (ACS) [3, 4]. Management varies and is critically important to follow institutional experience. Herein, we present our successful experience in treating refractory VF in a case of ST-elevation myocardial infarction (STEMI) with ES. We hope that sharing our experience in the management of this rare but potentially life-threatening phenomenon will help clinicians deal with such cases and reduce the in-hospital mortality of myocardial infarction(MI).

### Case presentation

**Fig. 1:**
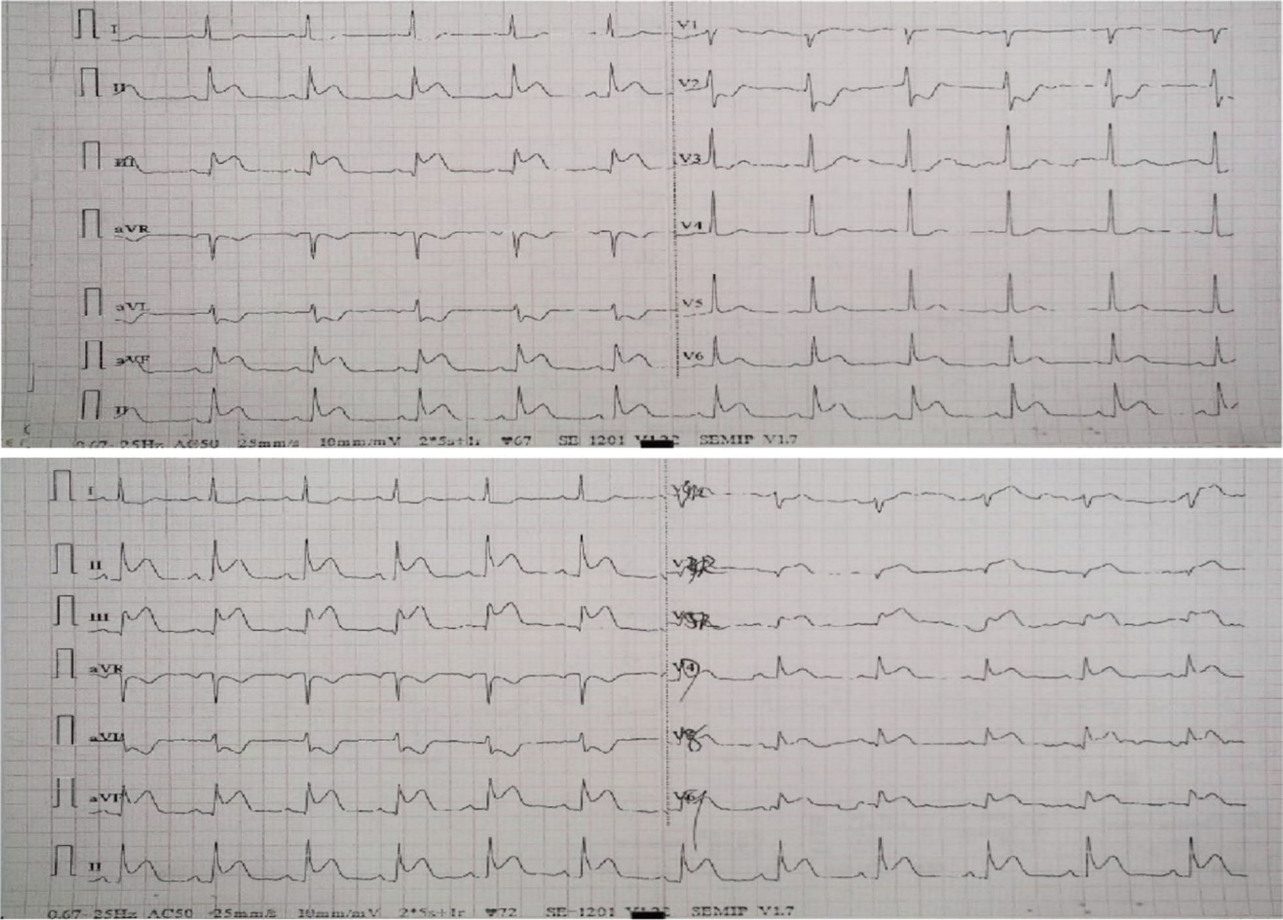
Admission 18-lead ECG showed sinus rhythm and ST-segment elevation in leads II, III, AVF, V3R-V5R, and V7-V9

A 57-year-old man presented to the emergency department approximately two hours after the onset of chest pain. His chief complaint was severe and persistent chest pain radiating to the back, near-death feeling with associated diaphoresis, pallor, and general fatigue. He denied dyspnea on exertion, abdominal pain, nausea, or vomiting. His past medical history includes uncontrolled hypertension and a 40-pack-year history of smoking. Importantly, there was no family history of MI or sudden death. Vitals on admission were temperature: 36.5 °C, pulse: 72 beats/min, respiration rate: 20/min, blood pressure: 137/87 mmHg (1 mmHg = 0.133 kPa), and oxygen saturation: 98%. Physical examination was unremarkable for the cardiovascular, gastrointestinal, respiratory, and nervous systems. A bedside standard 18-lead electrocardiogram (ECG) showed ST-segment elevation in leads II, III, AVF, V3R-V5 R, and V7-V9(Fig. 1). An impression of “acute infero-posterior ST-segment elevation MI complicated by acute right ventricular MI” was made, and dual antiplatelet therapy was initiated with 300 mg aspirin plus 180 mg ticagrelor. Initial laboratory tests were as follows: troponin I, 0.15 μg/L(reference range: 0.01 ~ 0.023 μg/L); creatine kinase isoenzyme MB, 31.7U/L(reference range: 0 ~ 20U/L); D-dimer, 258 μg/L(reference range: 80 ~ 500 μg/L); aspartate aminotransferase, 131U/L(reference range: 0 ~ 40U/L); alanine aminotransferase, 49U/L(reference range: 0 ~ 40U/L); C-reactive protein, 30.6 mg/dL(reference range: 0 ~ 10 mg/L); serum potassium, 3.72 mmol/L(reference range: 3.5 ~ 4.5 mmol/L); cholesterol levels, coagulation profile, renal profile, and N-terminal brain natriuretic peptide precursor were all normal.

**Fig. 2:**
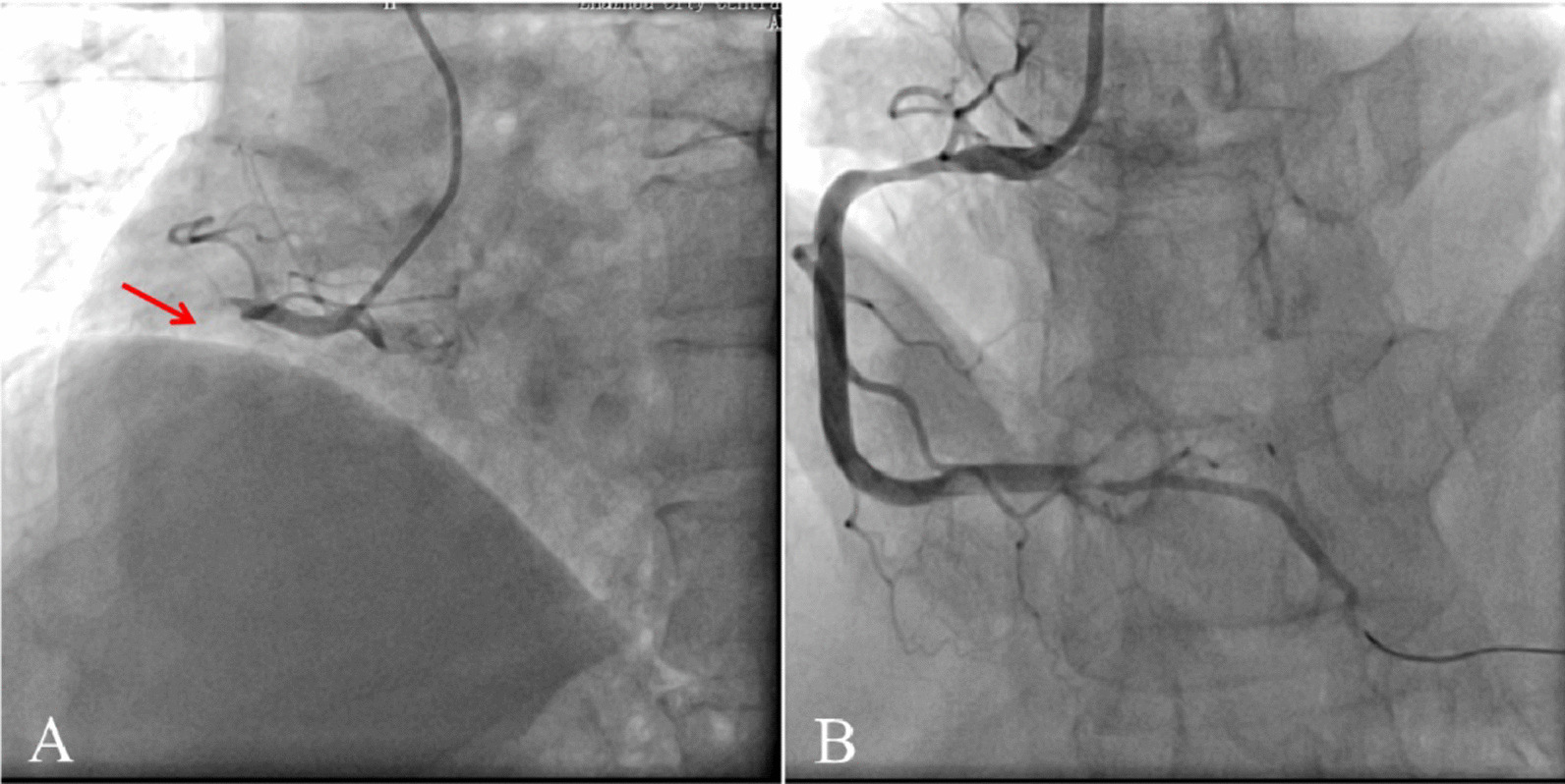
**A** Initial angigraph. Coronary angiogram using a guiging cather revealed totla occlusion of the proximal segment of the right coronary artery (red arrow). **B** Final angiography. Final coronary angogram showed TIMI-3 grade flow of the right coronary artery following stent implantion

As a major acute chest pain national referral center, the cardiac catheterization room was activated for planned emergency coronary angiography (CAG) and PCI after obtaining consent from the patient and the family. CAG showed right coronary dominance, single-vessel lesion, total occlusion of the proximal right coronary artery (RCA), and thrombolysis in myocardial infarction (TIMI) flow of grade 0 (Fig. 2A). Considering that the RCA was the occluded vessel and the importance of minimizing door-to-balloon (DTB) time, we performed PCI immediately following CAG.

**Fig. 3:**
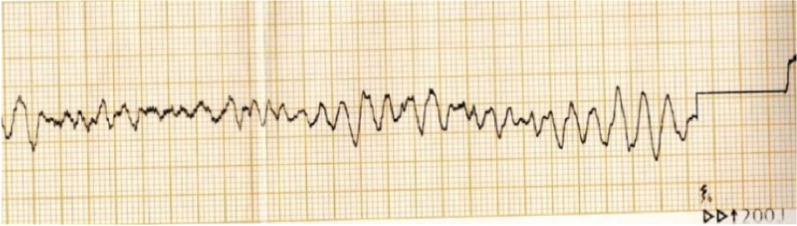
ECG at the onset of electrical strom

The patient suddenly lost consciousness when guiding the catheter into the RCA, and ventricular fibrillation (VF) ensued (Fig. 3). 200 J asynchronous electrical defibrillation was given immediately. Defibrillation successfully restored sinus rhythm. The second episode of VF was noted three minutes later, electric defibrillation was repeated, and 1 mg epinephrine was administered. One minute later, the third episode of VF occurred again, and a diagnosis of ES was considered. At the time of repeated electric defibrillation, multiple intravenous lines were running to maintain hemodynamic stability, electrolyte replacement (potassium and magnesium) was administered, and a loading dose of amiodarone at 150 mg intravenously followed by a maintenance dose of 1 mg/min. Esmolol was titrated at 0.5 mg/kg intravenously and maintained at 0. 05 ~ 0.20 mg/ (kg. min) through an infusion pump. Due to an impending airway compromise and patient anxiety and agitation, endotracheal intubation and mechanical ventilation were performed, and induced sedation was achieved with midazolam injection 0.01 mg/ (kg. h). Fourteen additional episodes of VF were recorded during the procedure before reverting to sinus rhythm. Vitals returned to baseline: HR: 62 bpm, BP: 108/69 mmHg, and when blood pressure dropped to 80/47 mmHg, the esmolol dosage was adjusted to 0.05 mg/ (kg. min) and norepinephrine 0. 2 ~ 0.3 μg/ (kg. min) pump was added for maintenance.

**Fig. 4:**
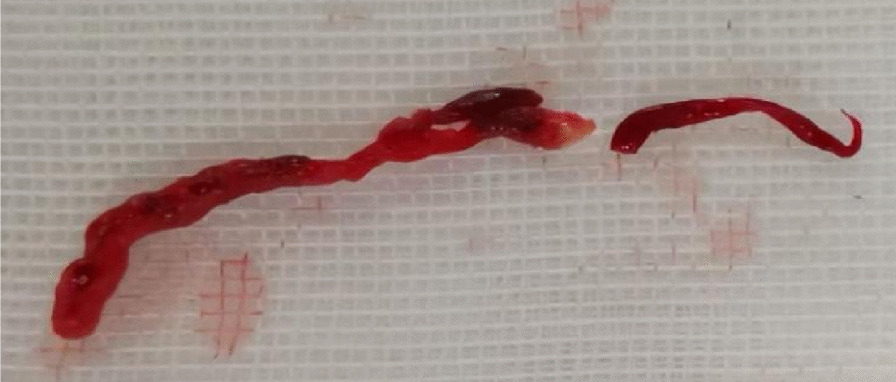
A successful capture of red thrombus during percutaneous coronary intervention

**Fig. 5:**
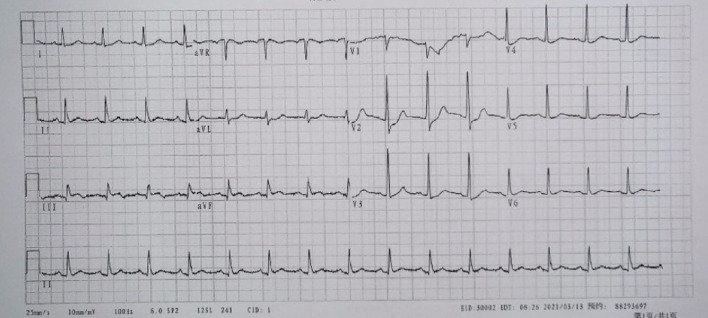
ECG after primary PCI showed resolution of ST-segment elevation

Percutaneous transluminal coronary angioplasty (PTCA) was done for the proximal RCA within a DTB time of 72 min. CAG showed multiple thrombi in the RCA, and the erythrocyte-rich thrombus was aspirated (Fig. 4). To reduce the thrombotic load, Tirofiban was administered intravenously with 10 μg/kg for 3 min and maintained at 0.15 μg/ (kg. min). Then a drug-eluting stent was implanted for RCA ostial narrowing. Stent deployment restored TIMI grade 3 flow (Fig. 2B). The patient was successfully transferred from the operating table to the cardiac care unit (CCU), and the ST-segment elevation of the patient was attenuated after the primary PCI (Fig. 5). Esmolol, midazolam, and norepinephrine pumps were discontinued after the blood pressure stabilized. Amiodarone was also discontinued owing to its side effects leading to prolonged QT interval.

Aspirin (100 mg once a day), ticagrelor (90 mg twice a day), pravastatin (20 mg once a night), metoprolol sustained-release tablets (47.5 mg once a day) were continued. Color Doppler echocardiography showed left atrium size (31 mm), left ventricle (40 mm), segmental right ventricular hypokinesis, and left ventricular ejection fraction of 60%. One week after admission, the patient was discharged without any symptoms and with an angiotensin-converting enzyme inhibitor(ACEI).

## Discussion and conclusions

Most ACS-related electrical storms (ES) occur in the anterior descending branch or proximal occlusion of RCA, often with cardiac insufficiency or low ejection fraction value, or after revascularization [5, 6]. Most of the arrhythmias that cause ES are variations of monomorphic ventricular tachycardia, but polymorphic VT and VF can also cause ES, and their clinical manifestations are variable [4, 7].

Acute management of ES includes identifying risk factors, reversing potential triggers, and restoring hemodynamic stability. Common causes of ES include acute ischemic events, electrolyte disturbances, worsening heart failure, past arrhythmia events, renal insufficiency, and poor medication compliance. Triggers for ES are not readily identifiable in most patients with structural heart disease [8]; however, evaluation for active myocardial ischemia should be strongly considered. Other triggers that significantly increase the electrical instability of the heart include electrolyte disturbances, acute congestive heart failure, arrhythmic side effects of antiarrhythmic drugs, infection, fever, thyrotoxicosis, hypoxia, drug intoxication, bradycardia-induced spontaneous tachyarrhythmias, and prolonged QT interval. Timely identification and management of these inducing factors is critical in the identification and management of ES [1, 9, 10].

In acute MI patients with VT or VF, if the pathological mechanism persists, it is mainly due to the excessive activation of the sympathetic nerve and abnormal increase of β receptor reactivity [3], leading to protracted VT or VF that can present as ES. The mechanism of VT or VF may be related to papillary muscle ischemia, or it may be caused by recurrent spasm of the coronary artery on the background of severe stenosis, leading to recurrent ischemia and reperfusion arrhythmias [11].

ES is an independent risk factor for cardiac death, with the highest risk of death (5.4 times) in the first three months [12]. Timely electrical defibrillation or cardioversion is the primary measure to restore hemodynamic stability in patients with ES. Still, excessive and frequent intervention can cause myocardial damage, calcium overload in myocardial cells, potassium loss, myocardial cell apoptosis, progressive heart failure, and aggravating arrhythmia [2]. Therefore, electrical defibrillation or cardioversion alone cannot be completely relied on, effective antiarrhythmic drugs should also be used. While lidocaine was not used in this case, it is the drug of choice for ES caused by MI. Conversely, it is ineffective when used in monomorphic VT [13] and has not been shown to prevent malignant ventricular arrhythmias in acute MI [14].

Amiodarone is a potassium channel blocker that reduces cardiac oxygen consumption, improves heart rate, and dilates coronary arteries. Most studies have indicated that amiodarone has an excellent therapeutic effect on patients with VT after AMI but is associated with QT prolongation as its major side effect. When a ventricular ES occurs, catecholamines in the body can increase 100–1000 times and can completely reverse the electrophysiological effects of class I and III antiarrhythmic drugs [15]. This partially weakens the effect of amiodarone in prolonging ventricular repolarization, making these antiarrhythmic drugs significantly less effective or even ineffective. Therefore, intravenous beta-blockers, as potential therapeutic agents to address the catecholamine surge characteristic of ES, have been considered the mainstay of antiarrhythmic drug therapy in such settings [16]. Furthermore, there is a growing body of evidence to support beta-blockers, including esmolol, in the setting of both cardiopulmonary resuscitation [17] and refractory VF [18]. β-receptor blockers block the action of sodium, potassium, and calcium ion channels, playing a central role in anti-arrhythmia by quickly reversing the excessive activation of the sympathetic nervous system, making the initially ineffective anti-arrhythmia drugs effective. Additionally, beta-blockers confer mortality benefits and reduce the incidence of VT [19, 20]. Amiodarone monotherapy is not effective against ES after MI compared to β-receptor blockers in combination with amiodarone [21]. Esmolol is an ultra-short-acting, selective β-blocker that inhibits β1 receptors by competing for catecholamine binding sites in the myocardium. The half-life of its distribution is only 2 min, and it is eliminated within 9 min. It has a rapid onset of action, short half-life, small toxicity, and side effects, and its effect can disappear quickly after withdrawal [22].

Implantation of an implantable cardioverter-defibrillator (ICD) in the acute phase of an ES is contraindicated; thus, the use of β-blockers and amiodarone is the cornerstone of ES after achieving hemodynamic stability[23]. Furthermore, studies have shown that early deep sedation combined with β-blockers is very effective in breaking the vicious cycle of sympathetic adrenergic overactivation[24]. Benzodiazepine sedatives have anti-anxiety, sleeping, muscle relaxation, and sedation effects, providing comfort and inhibiting sympathetic excitement caused by fear and anxiety, which can lead to sympathetic ES. Midazolam is a short-acting benzodiazepine with fast action and rapid metabolic inactivation[25].

Electrolyte supplementation is significant in saving sudden cardiac death caused by ES[26]. In this case, serum potassium was 3.72 mmol/L, lower than the recommended target potassium range after acute MI (4.5 mmol/L)[27]. In addition to supplementing potassium, the role of magnesium ions in antagonizing calcium ions and preventing potassium ion loss is paramount. The psychological effects of patients who are recipients of implantable cardioverter defibrillation should be considered, and appropriate educational and psychological interventions to improve outcomes for such patients should be considered[28].

If ES persists after these interventions, patients may be considered to benefit from more invasive options such as overspeed pacing therapy, early interventional ablation, intra-aortic balloon pump (IABP), and extracorporeal membrane oxygenation (ECMO)[8].

ES in acute MI is a severe but treatable clinical syndrome. A timely evaluation for its etiology is critical in order to quickly identify treatable causes, many of which benefit from early therapy. In order to get an appropriate management, it is necessary to track changes in ECG monitoring, judge and identify the risk factors of ES as soon as possible, and actively take early intervention measures for risk factors to avoid the occurrence of adverse events. Amiodarone combined with intravenous beta-blockers can be considered when repeated electrical defibrillation or cardioversion is less effective, which can well terminate the storm. In clinical practice, esmolol is recommended as one of the routine drugs in the treatment of severe ventricular arrhythmias and sympathetic ES.

## Data Availability

The datasets supporting the conclusions of this article are available in the GitHub repository, https://github.com/robin0336/Liubin.

## Abbreviations

ES: Electrical storm
VF: Ventricular fibrillation
VT: Ventricular tachycardia
MI: Myocardial infarction
STEMI: ST-segment elevation myocardial infarction
RCA: Right coronary artery
PCI: Percutaneous coronary intervention
ACS: Acute coronary syndrome
ECG: Electrocardiogram
CAG: Coronary angiography
TIMI: Thrombolysis in myocardial infarction
DTB: Door-to-balloon
PTCA: Percutaneous transluminal coronary angioplasty
CCU: Cardiac care unit
ACEI: Angiotensin-converting enzyme inhibitor
ICD: Implantable cardioverter-defibrillator
CRT: Cardiac resynchronization therapy
IABP: Intra-aortic balloon pump
ECMO: Extracorporeal membrane oxygenation
